# KS(conf): A Light-Weight Test if a Multiclass Classifier Operates Outside of Its Specifications

**DOI:** 10.1007/s11263-019-01232-x

**Published:** 2019-10-10

**Authors:** Rémy Sun, Christoph H. Lampert

**Affiliations:** 1 ENS Rennes, Bruz, France; 2 IST Austria, Klosterneuburg, Austria

**Keywords:** Multi-class classification, Specifications, Distribution shift, Deep convolutional networks

## Abstract

We study the problem of automatically detecting if a given multi-class classifier operates *outside of its specifications (out-of-specs)*, i.e. on input data from a different distribution than what it was trained for. This is an important problem to solve on the road towards creating reliable computer vision systems for real-world applications, because the quality of a classifier’s predictions cannot be guaranteed if it operates out-of-specs. Previously proposed methods for out-of-specs detection make decisions on the level of single inputs. This, however, is insufficient to achieve low false positive rate and high false negative rates at the same time. In this work, we describe a new procedure named KS(conf), based on statistical reasoning. Its main component is a classical Kolmogorov–Smirnov test that is applied to the set of predicted confidence values for batches of samples. Working with batches instead of single samples allows increasing the true positive rate without negatively affecting the false positive rate, thereby overcoming a crucial limitation of single sample tests. We show by extensive experiments using a variety of convolutional network architectures and datasets that KS(conf) reliably detects out-of-specs situations even under conditions where other tests fail. It furthermore has a number of properties that make it an excellent candidate for practical deployment: it is easy to implement, adds almost no overhead to the system, works with any classifier that outputs confidence scores, and requires no a priori knowledge about how the data distribution could change.

## Introduction

Over the last years, and in particular with the emergence of deep convolutional networks (ConvNets), computer vision systems have become accurate and reliable enough to perform tasks of practical relevance autonomously and over long periods of time. This has opened opportunities for the deployment of automated image recognition systems in many commercial settings, such as video surveillance, self-driving vehicles, and social media.

However, a major concern our society has about automatic decision systems is their reliability: if decisions are made by a trained classifier instead of a person, how can we be sure that the system works reliably now, and that it will continue to do so in the future? For other safety-critical software components, such as device drivers, static code analysis and formal verification techniques have been established to identify risks before even deploying the software. Unfortunately, such methods are still in their infancy for machine learning. Instead, quality control for trained system typically relies on extensive testing, making use of data that (a) was not used during training, and (b) reflects the expected situation at prediction time. If a system works well on a sufficiently large amount of data fulfilling both conditions, practical experience as well as statistical learning theory tell us that it will also work well in the future. We call this *operating within the specifications (within-specs)*.

In practice, problems emerge when a chance exists that the data distribution at prediction time differs from the one the creators of the classifier expected at training time, i.e. when condition (b) is violated. Such *operating outside of the specifications (out-of-specs)* can happen for a variety of reasons, ranging from user errors, over problems with the image acquisition setup, to unexpected objects occurring in the images, and even deliberate sabotage. Standard performance guarantees do not hold anymore in out-of-specs situations, and the prediction quality often drops substantially. This is irrespective of how well and on how much data a classifier was originally trained: even a system that works 100% accurately under within-specs conditions can produce predictions at chance level or worse when operating out-of-specs. Consequently, the out-of-specs problem has emerged as one of the major obstacles for deploying deep learning solutions for real-world applications.

Surprising as it is, today’s most successful image classification methods, multi-class ConvNets, are themselves not able to tell if they operate inside or outside the specifications. For any input they will predict one of the class labels they were trained for, no matter if the external situation matches the training conditions or not.

Clearly, it would be highly desirable to have an automatic test that can reliably tell when a given classifier operates out-of-specs, e.g. to send a warning to a human operator. Our main contribution in this work is such a test, KS(conf) , that is light-weight and theoretically well-founded, yet very powerful in practice. It builds on the observations that the confidence scores of a probabilistic classifier can be expected to change when operating out-of-specs. Intuitively, one would expect these changes not to be drastic enough to yield a test of sufficient quality (high true positive rate, low false positive rate), and this is indeed confirmed by a number of experiments that we report on. The main insight behind KS(conf) is that a more powerful test can be constructed by not judging individual samples, but batches of inputs together. Specifically, it compares the score distribution of predicted values to a reference set using a classical Kolmogorov–Smirnov test. The result is a simple and light-weight yet powerful test that is particularly well suited for practical use, because it works with arbitrary classifiers, including pretrained ConvNets. It also requires neither access to internal layers of the network nor a manipulation of the input images. By adjusting the batch size, the true positive rate can be improved without increasing the false positive rate at the same time. This is in contrast to single sample tests that only allow for a trade-off between both quantities.

Given the importance of the problem, in this work we put particular emphasis on a thorough experimental evaluation. We demonstrate the power of KS(conf) using five state-of-the-art ConvNets architectures (ResNet50, VGG19, SqueezeNet, MobileNet25, NASNetAlarge), challenging real-world image datasets (ImageNet ILSVRC 2012, Animals with Attributes 2, DAVIS) and a variety of possible out-of-specs scenarios (new classes, change of low-level image properties, loss of variability, problems in the image acquisition setup). To support other researchers testing the method for their own classifiers with their own data, we make our source code publicly available under a free and open-source license.

The rest of the manuscript is structured as follows: we first formalize the setting in Sect. 2 and formulate first principles that any practical test for out-of-specs operation should have. We then discuss existing work for out-of-specs detection in Sect. 3 and we highlight connections to related research areas. In Sect. 4 we describe our proposed method, including an analysis of its resource requirements. In particular, we show that it fulfills all the required criteria and also exhibits several additional useful properties. After an introduction to the experimental setting and data sources in Sect. 5, we present our experimental evaluation divided into three parts, each of which we consider of potentially independent interest: an analysis of the limits of tests acting on single samples for out-of-specs detection (Sect. 6), an analysis of batch-based methods (Sect. 7), and a study how modern ConvNets react to changes of their inputs acquisition setup. In Sect. 9 we discuss shortcomings of the proposed approach and provide an outlook on possible improvements. Finally, we conclude with a summary in Sect. 10.

## Testing for Out-of-Specs Operation

The task of testing for out-of-specs operation has appeared in different variants in the literature. In this section, we formally introduce the setting and define necessary criteria for tests to be applicable in real-world settings.

Throughout this work, we take the perspective of a computer vision system deployed in the real world in order to solve a practical task, such as classifying products in a store. The overarching goal is to determine whether the conditions under which the classifier operates at any time differ from the conditions for which it was created. Assuming a fully automatic classification system, the only relevant difference that can occur is a change in the input data distribution between training/validation and prediction time. Consequently, we define the goal of detecting out-of-specs operation as identifying such changes of the classifier’s input distribution, which we formalize in the following way:

### Definition 1

For a given classifier, let *X* denote its input, which we treat as a random variable with underlying distribution $P_X$. The classifier is said to *operate out-of-specs*, if the distribution, $P_X$, at prediction time differs from the one at training time.

In the rest of this section, we introduce some important properties that any test for out-of-specs operation should have. First, we ask for the test to be

- *passive:* The test should not influence the behavior of the classifier, even if an out-of-specs situation is detected.

The role of a passive test is to raise an alarm if a problem was detected, such that a human operator or expert can examine the problem and potentially resolve it. This is in contrast to active tests, that change the classifiers’ behavior, e.g. cause them to refuse to make predictions, or try to adapt them to the new conditions. While in other situations such active behavior might be desirable, in the context of this work we only consider passive systems, because we expect them to find easier acceptance by practitioners.

A second important constraint is that we expect out-of-specs conditions to occur rarely, maybe never during the lifetime of the system. Therefore, we particular care about the ability to avoid false alarms and ask tests to be

- *tunable:* The false positive rate (FPR) should be adjustable to any user-preferred level, ideally on-the-fly without interrupting the system’s operation.

In practice, reducing the false positive rate often comes at the expense of a lower true positive rate (TPR), i.e. more out-of-specs situations are missed, and the optimal setting reflects a task-dependent trade-off between both quantities. Studies have shown, however, that a too high false positive rate has a disproportionately negative effect: it will annoy the human operator, who then decides to ignore the alarms or switch off the test completely (Cvach 2012; Edworthy 1994; Häkkinen 2010).

A third crucial property is that any test that is meant to operate under real-world conditions needs to be

- *agnostic:* The test should not require a priori knowledge *how* the data distribution could change.

Unfortunately, this condition is violated in many tests that can be found in the literature, which assume that some data of the out-of-specs situations is a priori available. We want to avoid this, because in a real-world setting, the out-of-specs distribution is typically not known until it occurs, so the test must be able to capture any potential change.

Two more properties describe the classifiers to which a test is applicable. For highest practical usefulness, a test should be

- *universal:* The same test procedure should be applicable to different classifier architectures.
- *pretrained-ready:* The test should be applicable to pretrained and fine-tuned classifiers and not require any specific steps during training.

The first condition ensures that the same test remains useful, even when new classifier architectures emerge. The second condition reflects that practitioners typically do not have the resources or expertise to train a classifier from scratch, but prefer to rely on available pre-trained models.

Finally, in order to make a test as broadly applicable as possible, it should be

- *black-box ready:* The test should not require knowledge of any classifier internals, such as the depth, activation functions or weight matrices of a ConvNet, or access to intermediate computation results, such as a ConvNet feature layer.

This condition ensures that the test can be used with proprietary, e.g. commercial, classifiers, which typically do not reveal their inner working mechanisms.

## Related Work

A variety of methods have been proposed that aim at solving the problem of detecting out-of-specs operation or similar tasks. In this section, we discuss them in particular in light of the criteria we introduced in the previous section. A tabular overview of the properties of different methods can be found in Table 1.

**Table 1:** Overview of related methods and their properties

Method	Task	Granularity	Criteria					
			Passive	Tunable	Universal	Pretrained-ready	Black-box ready	Agnostic
Proposed: KS(conf)	Out-of-specs detection	Batch	✓	✓	✓	✓	✓	✓
Threshold-based test (Hendrycks and Gimpel 2017)	Out-of-specs detection	Single sample	✓	✓	✓	✓	✓	✓
ODIN (Liang et al. 2018)	Out-of-specs detection	Single sample	✓	✓	✓	✓	✗	✓/✗
OpenMax (Bendale and Boult 2016)	Out-of-specs detection	Single sample	✓	✗	✓	✗	✗	✓
Numerous methods, e.g. DeVries and Taylor (2018), Lakshminarayanan et al. (2017), Lee et al. (2018), Li and Gal (2017), Louizos and Welling (2017))	Out-of-specs detection	Single sample	✓	✓	✗	✗	✗	✓
Numerous methods, e.g. Knorr and Ng (1997), Tax and Duin (1998), Tax (2001)	Outlier detection	Single sample	✓	✓	✗	✗	✓/✗	✓/✗
Robust learning (Konstantinov and Lampert 2019)	Outlier detection	Dataset	—	—	✓	✗	✗	✓
Numerous methods, e.g. Bansal et al. (2014), Daftry et al. (2016), Scheirer et al. (2011), Zhang et al. (2014))	Failure prediction	Single sample	✓	✓	✓/✗	✓/✗	✓/✗	✗
Invariant feature learning, e.g. Ganin and Lempitsky (2015), Khosla et al. (2012), Long et al. (2015), Tommasi et al. (2012)	Domain adaptation	Dataset	✗	—	✓/✗	✗	✗	✗
Classifier adaptation, e.g. Patel et al. (2015), Wang and Deng (2018)	Domain adaptation	Dataset	✗	—	✓/✗	✓/✗	✗	✗
Class prior adaptation (Royer and Lampert 2015)	Domain adaptation	Batch	✗	—	✓	✓	✓	✗
Continual learning,e.g. Kirkpatrick et al. (2017), Rebuffi et al. (2016)	Handling unseen classes	Batch	—	—	✗	✗	✗	✗
Open set recognition, e.g. Bendale and Boult (2015), Jain et al. (2014)	Handling unseen classes	Dataset	—	—	✗	✗	✗	✗
Zero-shot learning, e.g. Akata et al. (2016),Palatucci et al. (2009),Lampert et al. (2014),Xian et al. (2018)	Handling unseen classes	Dataset	—	—	✗	✗	✗	✗
Numerous methods, e.g. Guo et al. (2017), Platt (1999)	Score calibration	Single sample	✓/✗	—	✓	✓	✓	✗
Numerous methods, e.g. Pollak (1985), Basseville and Nikiforov (1993)	Change point detection	Time series	✓	✓/✗	—	—	—	✓/✗
Numerous methods, e.g. Harel et al. (2014), Kuncheva and Faithfull (2014), Sethi et al. (2016), Wang and Abraham (2015), Zliobaite (2010)	Concept drift detection	Time series	✓	✓/✗	—	—	—	✓/✗

The task column indicates which problem the methods tries to solve. The granularity column specifies the type of input data (single samples, batches of samples, complete datasets, time series) that the methods take as input. For a definition of the criteria, see Sect. 2. As entries, ✓ indicates that a method has a certain property, ✗ indicates that it does not, ✓/✗ indicates that methods might or might not have this property, — indicates that this property does not apply in the situation

*Out-of-specs detection* A number of recent works have proposed methods for detecting data samples that derive from a different data distribution than expected. All of them implicitly rely on the assumption that these samples are atypical with respect to the original data distribution, i.e. they perform a form of outlier detection. For general remarks about this research direction, see our discussion below.

Most related to our setup, it has been reported in Hendrycks and Gimpel (2017) that modern multi-class ConvNets typically predict with lower confidence on data that is sampled from a data distribution different from the training data. Therefore, one obtains a simple test of out-of-specs behavior by raising an alarm when the confidence score of a data point falls below a threshold. This *threshold* classifier indeed fulfills all criteria we defined in Sect. 2. We discuss it in more detail in Sect. 6 and also provide an experimental comparison using more challenging image classification tasks than what was reported in the original works.

In Liang et al. (2018) it was observed that the difference in confidence scores between within-specs and out-of-specs operation grew when adding a *supportive perturbation* to the images and applying *temperature scaling* to the output. The resulting ODIN test requires access to the network internals, so it does not fulfill the criterion of being *black-box ready*. Nevertheless, we discuss and experimentally evaluate it in Sect. 6, again going beyond the original work by using state-of-the-art ConvNet classifiers and more challenging classification tasks.

Several further authors, e.g. DeVries and Taylor (2018), Lakshminarayanan et al. (2017), Lee et al. (2018), Li and Gal (2017), and Louizos and Welling (2017), and also a section of Hendrycks and Gimpel (2017), propose methods that require changing the classifier architecture, the training procedure, or the evaluation methodology to improve the ability of detecting if the data distribution changed. We do not discuss these further or evaluate them experimentally, because they fail to fulfill several of the core conditions, in particular of being *universal*, *pretrained-ready* and *black-box ready*. This means they cannot simply be used in combination with any given classifier.

*Outlier/anomaly/novelty detection* are classical tasks in unsupervised machine learning or data mining (Chandola et al. 2009; Hawkins 1980; Hodge and Austin 2004). They aim at identifying data points that are atypical in comparison to the bulk of the data in order to either remove them, or study them in more detail.

**Fig. 1:**
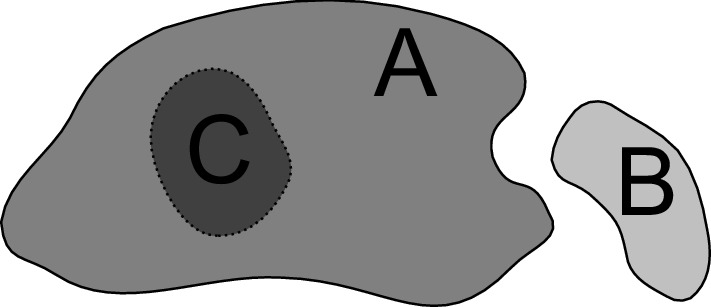
Illustration of the difference between *outlier detection* and *out-of-specs detection*. Let the region *A* reflect the data distribution at training time. Outlier detection methods are able to identify if at prediction time data from a new region, e.g. *B*, occurs. They are not able to identify if at prediction time only data from a subset of the training region occurs, e.g. only data from *C*, or no data from *C* at all. Out-of-specs detection aims at detecting all of these as well as any other change of data distribution

This is not the same problem as detecting an out-of-specs situation, but only a subset of it. Figure 1 illustrates the difference. Indeed, one possible out-of-specs scenario is when data samples of a type emerge that was not present at training time. For example, in a supermarket scenario an unknown product appears. Another out-of-specs situations, however, is when data of a type that is expected to occur fails to show up. For example, in a supermarket suddenly only cheap products are bought but no more expensive ones. A reliable out-of-specs detector would raise an alarm in both scenarios, while an outlier detector would only detect the first.

Classical outlier detection techniques work either probabilistically, e.g. by nonparametric *density estimation* (Knorr and Ng 1997), or geometrically, e.g. by *one-class classification* (Tax 2001). Neither approach is directly suitable for natural image data, though, because of the high data dimensionality and diversity of image data.

Recent work on *robust learning* (Konstantinov and Lampert 2019) aims at identifying outliers on the level of datasets instead of individual samples. This allows the use of unreliable data sources, but it happens at training time and does not address the problem of out-of-specs detection at prediction time.

*Failure prediction* Similar approaches as for outlier detection have been used to *identify failures*, i.e. predict when the label predicted by a classifier is incorrect (Bansal et al. 2014; Daftry et al. 2016; Scheirer et al. 2011; Zhang et al. 2014) and for *score calibration* (Guo et al. 2017; Platt 1999), i.e. adjusting the predicted scores to better reflect the probability of an error. These tasks are orthogonal to ours, as they concentrate on the situation where the network operates on data from the within-specs distribution, but nevertheless some predictions should not be trusted.

*Learning new classes* A specific out-of-specs situation is when new classes occur in the input data. Dedicated systems to handle this situation at training time have been suggested for *continual* (Kirkpatrick et al. 2017; Rebuffi et al. 2016) or *open set learning* (Bendale and Boult 2015; Jain et al. 2014). These methods operate at training time, though, and only for specific classifier architectures, therefore, they violate several of the relevant criteria of Sect. 2.

As an alternative, a threshold-based classifier was proposed in Bendale and Boult (2016). The authors introduce a score normalization procedure based on extreme-value theory. The exact method require access to the image features and the training set, though, so it is not *pretrained-ready* and not *blackbox-ready*.

For the case that new classes occur at prediction time and one still wants to classify them correctly, a variety of *zero-shot learning* (Xian et al. 2018) methods have been proposed, e.g. Akata et al. (2016), Lampert et al. (2014), Palatucci et al. (2009). All of these require specific classifier architectures and additional side-information about the class set, though, and are therefore not applicable to the situation we are interested in.

*Domain Adaptation/Transfer Learning* The study how classifier performance differs when the data distributions changes between training and prediction time and how this can be prevented is studied in the research area of *domain adaptation* (Ben-David et al. 2010). Typical techniques adjust the classifier training procedure to prevent a change in data distribution from negatively affecting the prediction quality (Patel et al. 2015; Wang and Deng 2018). One way to do this is by learning invariant features (Ganin and Lempitsky 2015; Khosla et al. 2012; Long et al. 2015; Tommasi et al. 2012) that ensure that even if the input data distribution changes in a certain way, the difference disappears after feature extraction. An alternative way is to learn explicit transformation between the features at training time and the features at prediction time, such that a mechanism is available for adapting the classifier to the new situation (Blitzer et al. 2006; Saenko et al. 2010). Both approaches, however, require some information about how the data distribution will change, at least in the form of unlabeled samples. If even a labeled dataset for the new distribution is available, *transfer learning methods*, such as *model fine-tuning* can be employed (Kuzborskij et al. 2013; Pan and Yang 2009). In all cases, the criterion of being *agnostic* to the out-of-specs conditions is violated, and often also the *black-box ready* and *pretrained-ready* criteria. An exception is (Royer and Lampert 2015), which post-processes the output scores at prediction time to match a new distribution. This, however, is not a *passive* technique, and it only works for a specific class of out-of-specs situations, namely changes in class priors.

*Time series analysis* The detection of changes in the data characteristics is also an important problem in time series analysis. In *change point analysis* (Basseville and Nikiforov 1993; Pollak 1985), the goal is to find the time points at which the characteristics of a time series make a substantial change. This differs from the problem we are interest in several ways. On the one hand, the problem has the additional difficulty that the position in the sequence where the change occurs is unknown. On the other hand, one typically deals with low-dimensional data, and stronger statistical techniques can be used because of the temporal structure of the data.

*Concept drift detection* (Gama et al. 2014) is another task in which one is interested in detected changes of a time series. In contrast to change point analysis, subtle changes are of interest, too. This is a hard task, and most existing methods are not directly applicable, because they require label annotations, e.g. Harel et al. (2014), Wang and Abraham (2015), or low-dimensional data, e.g. Kuncheva and Faithfull (2014), Sethi et al. (2016). An exception is (Zliobaite 2010), which even discusses the use of a Kolmogorov–Smirnov test. However, that is in the context of binary classifiers without a clear way to generalize the results to multi-class classification with large label sets.

## KS(conf) : Out-of-Specs Detection by Statistical Testing of Batches

In this section we introduce the proposed KS(conf) method for identifying when a classifier operates outside of the specifications.

We assume an arbitrary fixed multi-class classifier that, for any input, *X*, outputs a class label, *Y*, as well as a confidence score, *Z*, for its decision. For simplicity of discussion, we assume that the scores lie in the interval [0, 1], as it is the case for probabilistic systems, such as ConvNets with softmax output layer. Technically, this assumption is not necessary, though, as the method we describe only requires the scores to be bounded, i.e. lie in a finite interval, and that condition can always be achieved by a suitable, e.g. sigmoid, score transformation.

By our treatment of *X* as a random variable, *Z* also becomes a random variable with an induced probability distribution that we call $P_Z$. Analogously to the definition of *out-of-specs operation* in Sect. 2, we introduce the concept of *out-of-specs prediction*:

### Definition 2

A classifier is said to *predict out-of-specs*, if the output score distribution, $P_Z$, at prediction time differs from the one at training time.

Based on this nomenclature, we put forward the following hypothesis: testing for *out-of-specs prediction* can serve as an easy to implement and computationally light-weight proxy of testing for *out-of-specs operations*, provided that a suitable batch-based test is used. The last condition is important, because—as our experiments will show—existing tests based on single sample confidence scores are fundamentally limited in their ability to achieve a high true positive rate and a low false positive rate at the same time.

### Kolmogorov–Smirnov Test of Confidences

We propose a new method for out-of-specs testing that we call KS(conf) , which stands for *Kolmogorov-Smirnov test of confidences*. Its main component is the application of a Kolmogorov–Smirnov (KS) test (Massey 1951) to the distribution of confidence values, which at prediction time is estimated from batches of samples. KS(conf) has two main routines: *calibration* that is run once, and *batch testing* that is run continuously while the classifier is in operation.

*Calibration* In the calibration step, KS(conf) establishes a reference distribution that reflects the within-specs conditions. It is meant to be run when the classifier system is installed at its destination and a human expert is still present to ensure that the environment is indeed within-specs for the duration of the calibration phase.

**Fig. 2:**
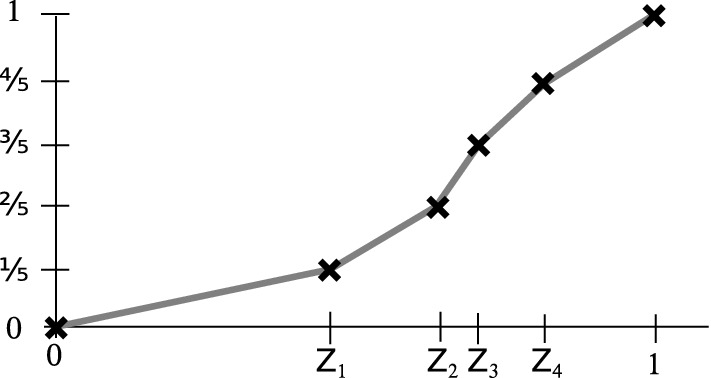
Illustration of (1): estimation of the cumulative distribution function (cdf) for $n=4$ data points. After sorting, each data point $Z_k$ is located at the $\frac{k}{n+1}$-quantile, i.e. the cdf has value $\frac{k}{n+1}$. Between the data points linear interpolation is used

To characterize the within-specs regime, we use the confidence scores, $Z^{\text {val}}_1,\dots ,Z^{\text {val}}_n$, of a set of validation images, $X^{\text {val}}_1,\dots ,X^{\text {val}}_n$. For simplicity we assume all confidence values to be distinct. In practice, this can be enforced by perturbing the values by a small amount of random noise. Because the *Z*-values are one-dimensional with known range, one could, in principle, estimate a probability density function (pdf) from a reasonably sized set of samples. For example, one would divide the interval [0, 1] into regular bins and count the fraction of samples falling into each of them. For our purposes, uniform bins would be inefficient, though, because classifier confidence scores typically concentrate at high values and are therefore far from uniformly distributed. To avoid a concentration of samples in a small number of bins with the remaining ones empty, one would have to resort to a data-adaptive estimation technique, e.g. data-dependent bins. KS(conf) avoids the need for this by starting with a pre-processing step. It estimates the *cumulative distribution function*, *F*, of the scores, which is possible without binning, see Fig. 2 for an illustration. First, one sorts the confidence values such that one can assume the values $Z^{\text {val}}_1,\dots ,Z^{\text {val}}_n$ in monotonically increasing order. Then, for any $p\in [0,1]$, the estimated cdf value at *p* is obtained by piecewise linear interpolation: for $k\in \{0,\dots ,n\}$ with $p\in [Z^{\text {val}}_{k}, Z^{\text {val}}_{k+1}]$,

1 $$\begin{aligned} F(p) = \frac{k}{n+1} + \frac{p-Z^{\text {val}}_k}{(n+1)\left( Z^{\text {val}}_{k+1}-Z^{\text {val}}_k\right) } \end{aligned}$$

with the convention $Z^{\text {val}}_0=0$ and $Z^{\text {val}}_{n+1}=1$.

The quantity that KS(conf) actually works with are normalized scores, $Z':=F(Z)$, where *F* remains fixed after calibration. By construction of *F*, the values $Z'$ will be distributed approximately uniformly in [0, 1], when *Z*’s distribution matches the distribution at calibration time. If the distribution of *Z* changes at a later time, this will be reflected by the distribution of $Z'$ differing from uniformity.

Besides removing the need for data-dependent (or any) density estimation, the above transformation is also useful for efficiency reasons: in the *batch testing* phase, we will not have to compare two arbitrary distributions to each other, but only the currently observed distribution with the uniform one.

**Fig. 3:**
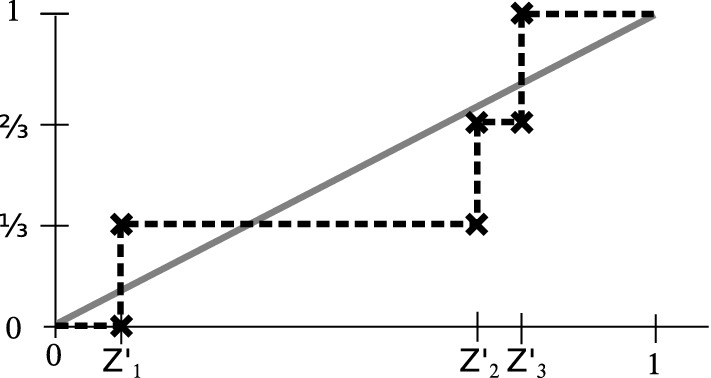
Illustration of (2): KS statistics for $m=3$ data points. To identify the biggest absolute difference between the empirical cdf (dashed line, black) and the uniform target cdf (solid line, gray), it suffices to check the difference between the two curves at the location of the data points. After sorting, at any $Z'_k$ the empirical cdf jumps from $\frac{k-1}{m}$ to $\frac{k}{m}$, while the target cdf has the value $Z'_k$

*Batch testing* The main step of KS(conf) is *batch testing*, which identifies if the system currently predicts within-specs or out-of-specs. This step it meant to be run repeatedly at the classifier’s operation time, i.e. after the system has been activated to perform its actual task.

The batches of images, $X_{1},\dots$, $X_{m}$, used for testing can, but do not have to, coincide with the image batches that are often used for efficient classifier evaluation on parallel architectures such as GPUs. A possible real-word scenario would be that batch testing is run at regular intervals, e.g. once per hour for applications that are not time-critical.

The actual test consists of the following steps. First, one applies the cdf that was learned during calibration to the confidence scores, resulting in values, $Z'_{1},\dots ,Z'_{m}$. As above, we treat these as sorted. Then, one computes their Kolmogorov–Smirnov (KS) test statistics

2 $$\begin{aligned} \textsf {KS} := \max \left( \max _{k=1,\dots ,m}\left\{ Z'_{k} - \frac{k-1}{m} \right\} , \max _{k=1,\dots ,m}\left\{ \frac{k}{m} - Z'_k \right\} \right) . \end{aligned}$$

$\textsf {KS}$ measures the largest absolute difference between the empirical cdf of the observed batch and a linear increasing reference cdf, see Fig. 3 for an illustration. For a system that operates within-specs (and therefore predicts within-specs), $Z'$ will be close to uniformly distributed, and $\textsf {KS}$ can be expected to be small. It will not be exactly 0, though, because of finite-sample effects. A particularly appealing property of the $\textsf {KS}$ statistic is that its stochastic fluctuations are well understood and confidence thresholds for the finite-sample situation have been derived (Massey 1951). This yields the Kolmogorov–Smirnov test: for any $\alpha \in [0,1]$ there is a threshold $\theta _{\alpha }$, such that when we consider the test outcome *positive* for $\textsf {KS} > \theta _{\alpha }$, then the expected probability of a false positive test result is $\alpha$. The values $\theta _{\alpha }$ can be computed numerically (Marsaglia et al. 2003) or approximated well (in the regime $n\gg m$ that we are mainly interested in) by $\theta _{\alpha }\approx (\frac{-0.5\log (\frac{\alpha }{2})}{m})^{\frac{1}{2}}$. A list of tabulated values can be found in Table 10.

The Kolmogorov–Smirnov test has several advantages over other tests. Importantly, it is *distribution-free*, i.e. the thresholds $\theta _{\alpha }$ are the same regardless of what the distribution $P_{Z}$ is. Also, it is invariant under reparameterization of the sample space, which in particular means that the $\textsf {KS}$ statistics and the test outcome we compute when comparing $Z'$ to the uniform distribution are identical to the one for comparing the original *Z* to the original within-specs distribution. This fact implies that KS(conf) will not be negatively affected by classifiers that produce overly confident outputs, and that KS(conf) is compatible with and invariant to potential classifier score calibration techniques. Finally, the Kolmogorov–Smirnov test is known to have asymptotic power 1, meaning that if given enough data, it will detect any possible difference between distributions.

While one could imagine constructing tests based on other measures of similarity between distributions, these generally do not share the advantageous properties of KS(conf) . For example, *total variation* distance requires density estimation and can therefore only be approximated, not computed exactly. It is also not invariant under reparametrizations of the scores. *Kullback-Leibler* or *Jensen-Shannon divergence* cannot reliably be estimated at all from finite sample sets, unless one makes additional assumptions about the underlying distributions. Furthermore, they might take infinite values.

**Table 2:** Details of the ConvNets used for the experimental evaluation

Network name	ILSVRC2012 error		Number of parameters (M)	Speed: GPU			CPU
	Top-1 (%)	Top-5 (%)		$\textsf {bs} =1$ (ms)	$\textsf {bs} =10$ (ms)	$\textsf {bs} =100$ (ms)	$\textsf {bs} =1$ (ms)
MobileNet25 (Howard et al. 2017)	48.4	24.2	0.48	3.3	5.2	34	682
SqueezeNet (Iandola et al. 2016)	45.6	21.4	1.2	5.7	10.1	113	2288
ResNet50 (He et al. 2016)	25.1	7.9	26	12.2	34.1	293	–
VGG19 (Simonyan and Zisserman 2014)	28.7	10.2	144	9.9	53.5	385	–
NASNetAlarge (Zoph et al. 2018)	17.5	3.9	94	45.8	227.9	2107	–

Evaluation time (excluding image preprocessing and network initialization) for different batch sizes ($\textsf {bs})$ on powerful GPU hardware (NVIDIA Tesla P100) or weak CPU hardware (Raspberry Pi Zero). Missing entries are due to memory limitations

*Properties and resource requirements* A quick check of its properties shows that KS(conf) fulfills all criteria for a practical test that we introduced in Sect. 2. Furthermore, it can be implemented in a straight-forward way and requires only standard components, such as sorting and linear interpolation. The largest resource requirements occur during *calibration*, where the network has to be evaluated for *n* inputs and the resulting confidence values have to be sorted. The *calibration* step is performed only once and offline though, before actually running the classification system under real-time conditions. Therefore, $O(n\log n)$ runtime is not a major problem, and even very large *n* remain practical. A potential issue is the *O*(*n*) storage requirements, if calibration is meant to run on very small devices or very large validation sets. Luckily, there exist specific data structures that allow constructing approximate cdfs of arbitrary precision in an incremental way from streaming data, for example, *t-digest*s (Dunning and Ertl 2014).

The *batch testing* step runs during the standard operation of the classification system and therefore needs to be as efficient as possible. Implemented as described above, it requires applying the cdf function to every sample, which typically would be done by an $O(\log n)$-binary search. Subsequently, the *m* confidence values need to be sorted, and the maximum out of 2*m* values identified. Overall, the runtime complexity is at worst $O(m\log n)$ and the memory requirement is *O*(*m*).

In summary, with only logarithmic overhead, KS(conf) ’s computational cost is negligible compared to evaluating the classifier itself. For even more restricted settings one could rely on incremental variants of the Kolmogorov–Smirnov test, e.g. dos Reis et al. (2016).

## Experiments: Overview

In the following sections we report on a variety of experiments that compare different methods for out-of-specs detection, including KS(conf) , and provide an in-depth analysis of their success and failure cases. Given the large number of different scenarios, we only provide summary results and highlight specific cases that we consider of specific interest. A complete set of results as well as source code for their reproduction is available on the accompanying website.^1^

### Experimental Setup

With our objective of practical usefulness in mind, we aim in our experiments for results that can be expected to generalize also to future image classification systems. We therefore emphasize three aspects in our experiments: (1) tackling a challenging task of high-resolution natural image classification; (2) obtaining results for a diverse set of ConvNet classifiers that constitute the state-of-the-art in different application scenarios; (3) working under as realistic conditions as possible, in particular not making use of information that is not available for real-world systems. This focus constitutes a major difference to many existing works that provide results on simpler datasets, such as MNIST or CIFAR, benchmark only few and relatively small network architectures, or adjust hyper-parameters on out-of-specs data.

Specifically, we reports results for five popular ConvNet architectures: ResNet50 (He et al. 2016) and VGG19 (Simonyan and Zisserman 2014) are standards in the computer vision community; SqueezeNet (Iandola et al. 2016) and MobileNet25 (Howard et al. 2017) have smaller computational and memory requirements, making them suitable, e.g., for mobile and embedded applications; NASNetAlarge (Zoph et al. 2018) achieves state-of-the-art performance in the ImageNet challenges, but is quite large and has high computational requirements. Technical details of the networks are given in Table 2.

All classifiers are pretrained on the training part of the ImageNet ILSVRC 2012 dataset (Russakovsky et al. 2015) (1.2 million training images of 1000 classes), which is the dataset most commonly used for this purpose.^2^ We use the 50.000 validation images of the same dataset as reference set for within-specs behavior. We do not make use of ground truth labels of the validation set (or the test set) at any time, as those would not be available for actually deployed systems, either.

At prediction time, for within-specs operation we use the 100,000 images of the ILSVRC 2012 test set as data source. For out-of-spec operation, we study three situations: (1) images with different contents than what is present in ILSVRC, e.g. new object classes; (2) images with the same contents as ILSVRC but different low-level characteristics that could, e.g., be caused by image acquisition problems; and (3) images with of the same type as ILSVRC, but occurring repeated over time, i.e. “frozen” images as they can occur during image transmission failures.

As additional data sources, we use the 10 test classes (proposed split) of the *Animals with Attributes 2 (AwA2)* dataset (Xian et al. 2018). These are 7913 natural images of similar appearance as ILSVRC, but showing classes that are not present in the larger dataset. Additionally, we also use the 3456 images from the DAVIS (Perazzi et al. 2016) dataset (*480p* part). Those images are in fact video frames and therefore also exhibit different characteristics than the still images of ILSVRC, such as motion blur.

To simulate repeating images, for each *test* image we form virtual data sets that consist of as many copies of that images as currently required. Results reported for this setting are always average values over all tested images.

In Sect. 8, where we provide a detailed assessment how state-of-the-art network react to potential problems in the image acquisition, we create images with synthetic distortions, such as added noise or blur. These are described in detail in the corresponding sections.

## Experiments: Out-of-Specs Detection from Single Samples

**Fig. 4:**
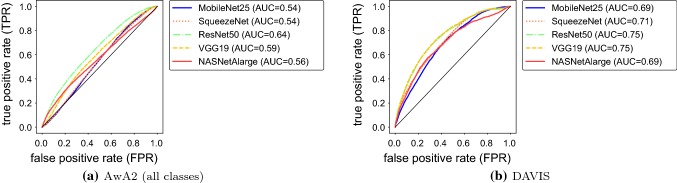
ROC curves of a threshold-based classifier based on confidence scores of five ConvNets classifiers for two data sources. AUC denotes the area under the ROC curve. One can see that the DAVIS out-of-specs situations is generally easier to detect than the AwA2, but none of the classifiers is able to achieve high TPR and high FPR at the same time

**Fig. 5:**
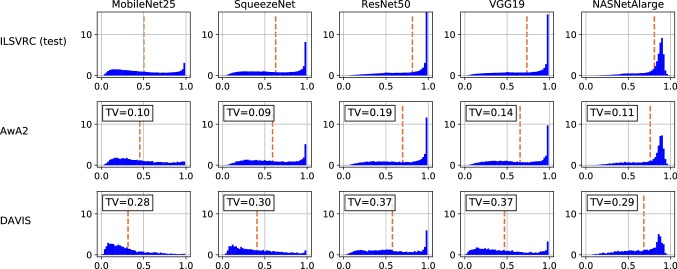
Distribution of confidence scores (*x*-axis) for different ConvNets and data sources. The dashed line indicates the average score. The TV value is the estimated *total-variation distance* between the plotted distribution and the corresponding within-specs situation

Previous work on detecting if a classifier operates on unexpected data, in particular (Hendrycks and Gimpel 2017; Liang et al. 2018), act on individual data samples. At prediction time, for every input image a separate decision is made whether it stems from a within-specs or out-of-specs situation. The experimental evaluation was limited to rather small networks and image sizes, though. In this section, we repeat and extend these single-sample experiments in the more challenging and diverse situation that we are interested in.

Following (Hendrycks and Gimpel 2017) we test a threshold-based detector that classifies a sample as out-of-specs if its confidence score lies below a threshold. By varying the threshold and recording the false positive as well as the true positive rate, one obtains a *receiver operating characteristic (ROC)* curve, one for each classifier and each out-of-specs situation.

Figure 4 shows the curves for the out-of-specs case where the classifier’s input is the AwA2 (all classes) or DAVIS data. Also listed the *area under the ROC curve* (AUC) values, which corresponds to the probability that a randomly selected within-specs samples has a higher confidence score than a randomly selected out-of-specs sample. In order to better understand the limitations of detecting out-of-specs situations from single samples, we visualize the actual score distribution in Fig. 5 and include an estimate of the total-variation distances between the out-of-specs situations and the respective within-specs ones.

We do not include separate experimental results for the case when the out-of-specs condition is caused by repeating images, because for single-sample tests these can be deduced from other experiments: for ILSVRC-test, single-sample tests are inherently unable to distinguish the scenario of repeating images from the within-specs one. Consequently, the best true positive rate is identical to the false positive rate, i.e. chance performance, and the AUC is 0.5. For AwA2 and DAVIS, the average AUC over all images is identical to the values in Fig. 4.

### Discussion of Results

From Fig. 4 one can see that overall the DAVIS situation is easier to detect than AwA2, but in neither case and for none of the ConvNets a threshold-based classifier is a strong tool for out-of-specs detection.

From Fig. 5 we can understand the reasons. The first row reflects within-specs behavior. It confirms the folk wisdom that ConvNet scores are biased towards high values. However, it also shows remarkable variability between different networks. For example, MobileNet25 has a rather flat distribution compared to, e.g., VGG19, and the distribution for NASNetAlarge peaks not at 1 but rather at 0.9. The other two rows show that out-of-specs operation indeed leads to a change of score distribution. The effect is not as strong as one might have expected, though, and in particular, there is no drastic shift of confidence scores towards very small values. The estimated total-variation distances between the out-of-specs situations and the respective within-specs ones quantify this effect. In particular, the TV value provides a theoretical upper limit on how well any single-sample test can distinguish between two distributions, even if it had access to perfect information about the distributions. Formally, the following two statements hold:

#### Theorem 1

1. For any single-sample test (not only threshold-based), the difference between true positive rate and false positive rate cannot be larger than $\textsf {\textsf {TV}}$.
2. No threshold-based test can achieve an AUC higher than $\frac{1}{2}(1+2\textsf {\textsf {TV}}-\textsf {\textsf {TV}}{\,}^2)$.

The first statement was proved in Kailath (1967) with correction in Toussaint (1972). The second statement follows from the implied upper bound to the ROC curve.

To illustrate the result, we look at AwA2, in which images have similar low-level characteristics as in the within-specs situation and $\textsf {TV} <0.2$ for all classifiers. The theorem implies that none of the ConvNets would allow a single-sample test that achieves a TPR more than 0.2 higher the FPR, and the AUC of a threshold-based test cannot exceed 0.68. Indeed, this analysis is consistent with the results in Fig. 4. Unfortunately, for practical applications these values are clearly insufficient.

### Results with Pre- and Post-Processing

A potential remedy for above problem could be image pre-processing and score post-processing, e.g. like the ODIN procedure in Liang et al. (2018). There it was reported that the quality of a threshold-based detector could be improved drastically by adding a supportive perturbation to the image before classifying it and post-processing the scores by temperature scaling. By adopting these steps the resulting test is not *blackbox-ready* anymore, because computing the supportive perturbations requires access to the network’s internal structure and parameters.

For completeness, we evaluated the tests anyway, following the description and source code of Liang et al. (2018). We provide a summary of results here, more details can be found as part of the accompanying website. Table 3 shows the highest AUC score achieved for any choice of temperature and strength of supportive perturbation on the AwA2 and DAVIS data. One can see certain improvements over a threshold-based test without pre- and postprocessing, as reported in Fig. 4. However, they are smaller than the ones reported in Liang et al. (2018). In particular, as a test of out-of-specs prediction, the quality is still far from sufficient for real-world problems. Consequently, we do not make use of pre- or postprocessing in the rest of this work. However, if desired, ODIN or any other image preprocessing and score post-processing techniques could readily be combined with KS(conf) or any other *universal* test that only requires confidence scores as input.

**Table 3:** Detection quality (AUC) of a threshold-based test with ODIN pre-/postprocessing

	AwA2	DAVIS
MobileNet25	0.58 (+0.04)	0.74 (+0.05)
SqueezeNet	0.54 (0.00)	0.76 (+0.05)
ResNet50	0.71 (+0.07)	0.80 (+0.05)
VGG19	0.60 (+0.01)	0.79 (+0.04)
NASNetAlarge	0.62 (+0.06)	0.69 (0.00)

The values in brackets indicate the difference to the plain test, as in Fig. 4. ODIN’s hyperparameters are chosen to maximize detection quality, i.e. not agnostically. Nevertheless, the improvements are rather limited

## Experiments: Out-of-Specs Detection from Batches

In this section, we report on an experimental evaluation of batch-based tests for detecting out-of-specs operation. Besides KS(conf) , we test a variety of alternative ways for combining the set of confidence scores in the batch and how to come up to a within-specs or out-of-specs decision. Specifically, we include the following tests as baselines:

*Mean-based tests* We saw in Sect. 6 that on average, the confidence scores are in fact lower in the out-of-specs situation than within-specs, but that the high variance prevents single-sample test from being reliable. The use of batches allows reducing the variance, which suggests a straight-forward test for out-of-specs behavior: for a batch of images, compute the average confidence and report a positive test if that value lies below a threshold.

To set the threshold, we have two options:

- *z-test* We compute the mean, $\mu$, and variance, $\sigma ^2$, of the confidence values on the validation set. Under an assumption of Gaussianity, the distribution of the average confidence over a within-specs batch of size *m* will have variance $\sigma ^2/m$. We set the threshold to identify the lower $\alpha$-quantile of that Gaussian.
- *(non-parametric) mean test* To avoid the assumption of Gaussianity, we use a bootstrap-like strategy: we sample many batches from the validation set and compute the mean confidence for each of them. The threshold is set such that at most a fraction $\alpha$ of the batches is flagged as positive.

The *z*-test has the desirable property that the threshold can be adapted on-the-fly even after the test has already been deployed. The mean test can be expected to work better for small batches, where the assumption of Gaussianity is likely violated, but any change of threshold will require a new step of bootstrapping, which, in particular, requires access to the previously confidence values or to new validation data.

**Fig. 6:**
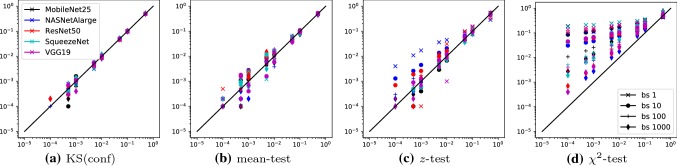
False positive rates of KS(conf) and three baselines across five different classifiers (colors) and different batch sizes ($\textsf {bs}$, symbols). The *x*-axis indicates the target FPR, the *y*-axis indicates the tests’ actual FPR. For an ideal test all entries should lie on the diagonal or below with no discernable pattern in the deviations. KS(conf) and the mean-test achieve this, while the *z*-test and the $\chi ^2$-test produce more false positives than targeted, in particular for small batch sizes (Color figure online)

*Probabilistic tests* Assuming probabilistic classifier outputs, the right way of combining scores within a batch is by multiplying them, or equivalently, averaging their logarithms. Doing so yields two tests, $\log$-*z* and $\log$-mean that follow the same steps as the *z*-test and the mean-tests, respectively, but work with the logarithms of the confidence values.

*Symmetric tests* The four tests described above are asymmetric: they will detect if the confidences become too low, but not if they become too high. To cover that possibility, we also include symmetric versions of the above tests, for which we determine two thresholds, an upper and a lower one, allowing for $\alpha /2$ false positives on each side.

*Label-based test* Instead of using the confidence values, it would also be possible to detect out-of-specs behavior from the distribution of actually predicted labels.

- $\chi ^2$*test* During calibration, we compute the relative frequency of labels on the validation set. For any batch, we perform a $\chi ^2$ goodness-of-fit test, whether the empirical distribution is likely to originate from the stored one and report a positive test if the *p*-value lies below the desired FPR.

### Results: False Positive Rates

As discussed in Sect. 2, it is a crucial property of a practical test to have a controllable false positive rate. We check this property for KS(conf) and the other batch-based tests by the following procedure: for any batch size $$\text {\textsf {bs}}\in \{1,10,100,1000\}$$ and $$\alpha \in \{0.0001,0.0005,0.001,0.005,$$$$0.01,0.05,0.1,0.5\}$$ we set the test’s parameter for a target FPR of at most $$\alpha $$. Then we run the test on batches sampled randomly from the ILSVRC test set, i.e. fully under within-specs conditions. Consequently, all positive tests are false positives and the fraction of tests that return positively is the FPR.

**Table 4:** True positive rates, averaged across 5 ConvNets, of KS(conf) and baselines (columns) under different out-of-specs conditions (rows)

	KS(conf)	Mean	Log-mean	Sym. mean	Sym. $\log$-mean
*Diverse*					
AwA2 (all classes)	1.00	**0.99**	**0.85**	**0.97**	**0.80**
DAVIS	1.00	1.00	1.00	1.00	1.00
*Specific*					
AwA2-bat	1.00	1.00	1.00	1.00	1.00
AwA2-blue-whale	1.00	$\underline{\mathbf{0.00 }}$	$\underline{\mathbf{0.00 }}$	**0.43**	$\underline{\mathbf{0.00 }}$
AwA2-bobcat	1.00	$\underline{\mathbf{0.00 }}$	$\underline{\mathbf{0.00 }}$	1.00	$\underline{\mathbf{0.00 }}$
AwA2-dolphin	1.00	1.00	**0.60**	1.00	**0.60**
AwA2-giraffe	1.00	1.00	1.00	1.00	1.00
AwA2-horse	1.00	1.00	1.00	1.00	1.00
AwA2-rat	1.00	1.00	1.00	1.00	1.00
AwA2-seal	1.00	$\underline{\mathbf{0.00 }}$	$\underline{\mathbf{0.00 }}$	1.00	$\underline{\mathbf{0.00 }}$
AwA2-sheep	1.00	$\underline{\mathbf{0.00 }}$	$\underline{\mathbf{0.00 }}$	**0.80**	$\underline{\mathbf{0.00 }}$
AwA2-walrus	1.00	1.00	1.00	1.00	1.00
*Static*					
ILSVRC (test)	1.00	**0.40**	**0.34**	**0.96**	**0.96**
AwA2 (all classes)	1.00	**0.51**	**0.44**	**0.96**	**0.44**
DAVIS	1.00	**0.71**	**0.65**	**0.97**	**0.65**

For all tests, the target FPR is set to 0.01 and the batch size to 1000. Test that fail occasionally ($\text {TPR}<1$) are marked in bold, tests that fail completely ($\text {TPR}=0$) in bold underlined

Exemplary results are depicted in Fig. 6, where we report the average FPR of four of the tests. The remaining results are summarized below and can be found in the accompanying website. Each plot contains 160 measurements: one for each combination of 5 classifier (encoded in color), 4 batch sizes (encoded in symbols) and 8 target FPRs (encoded by *x*-coordinate). The *y*-coordinate shows the actually measured FPR, averaged over 10,000 repeats. For an ideal test, all points should lie on the diagonal or below.

One can see that KS(conf) and the *mean*-test respect the FPR rather well. For KS(conf) , this is expected, as the underlying Kolmogorov–Smirnov test has well understood statistics and optimal thresholds are known for any FPR and batch size. For the *mean*-test, the reason is that the thresholds were obtained by simulating the testing procedure on within-specs data many times. This is computationally costly, especially for large batch sizes, but it ensures that the FPR is respected, as long as the validation set size is large enough. The same outcomes also hold for the $\log$-*mean* test and the symmetric variants of both tests, which are not visualized here.

In contrast, the *z*-test often produces more false positives than intended, especially for small batch sizes. This is an indication that the assumption of Gaussianity, which is violated for small batch sizes, actually matters in practice. While not depicted here, the $\log$-*z* test and the symmetric variants have the same problem as the *z*-test.

Finally, the label-based $\chi ^2$-test produces far too many false positives. The likely reason for this is the large number of classes: a rule of thumb says that the $\chi ^2$-test is reliable when each bin of the distribution has at least 5 expected entries. This criterion is clearly violated in our situation, where the number of samples in a batch is often even smaller than the number of classes (which is the number of bins).

In summary, of all methods, only KS(conf) achieves the two desirable properties that the FPR is respected for all batch sizes, and that adjusting the thresholds is possible efficiently and without access to validation data. Tests based on averaging the scores or logarithms of the scores should only be used with the bootstrap-based procedure for threshold selection. The *z*-approximation as well as the $\chi ^2$-based test are not reliable enough for practical use, so we exclude them from further experiments.

### Results: True Positive Rate

The ultimate quality measure for any test is whether, at a fixed FPR, it can reliably detect if the input distribution changes in any way. For this, we run the different batch-based tests on different out-of-specs situations: as a *diverse* scenario, we use the classifiers to make predictions on a mixture of all AwA2 classes or on the DAVIS data. As a *specific* scenario, we use each of the AwA2 classes individually as out-of-specs data source. Finally, as *static* scenario, we use all ILSVRC-test data, AwA2 data or DAVIS data, but the classifiers run on static data, i.e. each batches consists of multiple copies of the same image.

The first two cases correspond to a situation in which the classifier operates unperturbed, but on unexpected data. The third case reflects the situation where the input to the classifier is perturbed, e.g. because of network problems or by explicit manipulation. Note that it can still be interpreted as change in data distribution, only that the distribution at prediction time is a delta-peak on a single image.

For the *diverse* and *specific* situations, we compute the fraction of positive tests out of randomly created 10,000 batches. For the *static* situation, we create one batch for each image of the respective dataset and report the averages. In all cases, the system runs completely out-of-specs. Therefore, all positive tests are correct and reported averages directly correspond to the TPR.

**Fig. 7:**
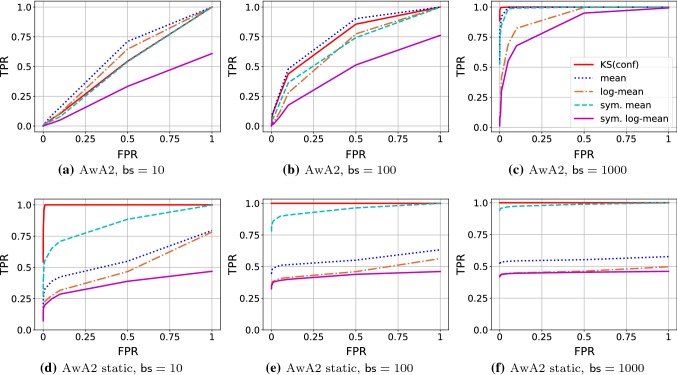
Exemplary curves of true positive rates (TPR, *y*-axis) versus target false positive rates (FPR; *x*-axis) for SqueezeNet on *diverse* AwA2 data (top row) and *static* AwA2 data (bottom row) with different batch sizes, $\textsf {bs}$. Note that the curves differ slightly from typical ROC-curves because the *x*-axis shows the tests’ target FPR, not a measured one. In particular, this means that the TPR might not reach a value of 1 even for $\text {FPR}=1$. For a discussion of the results, see Sect. 7.2

**Fig. 8:**
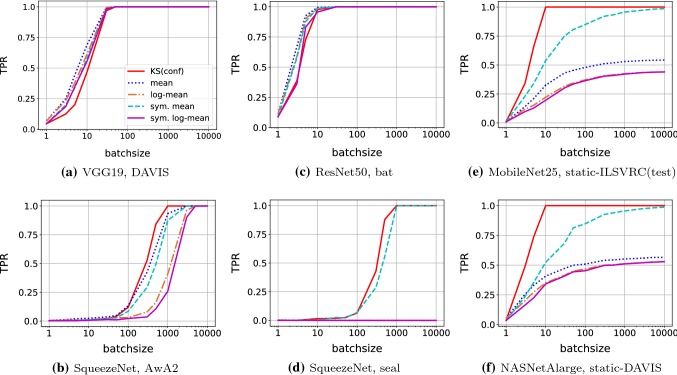
Exemplary curves of true positive rates (TPR, *y*-axis) for different tests run with different batch size (*x*-axis) on different out-of-specs data with $\text {FPR}=0.01$. For a discussion of the results, see Sect. 7.2

Because of the large number of scenarios and results, we only report on some characteristic cases and unexpected findings. The full set can be found in the accompanying website. Specifically, we first provide a quantitative summary (Table 4) and we then discuss the qualitative dependence between TPR and FPR (Fig. 7), between TPR and batch size (Fig. 8).

*Quantitative Summary*   Table 4 summarizes the results numerically at a single glance. It reports averaged TPRs across the five ConvNets for batch size 1000 and FPR 0.01.

It shows that in the *diverse* setting with all AwA2 classes or the DAVIS dataset as out-of-specs data, KS(conf) , the mean test and, to a lesser degree, the symmetric mean test do a good job detecting the change of distribution. The logarithmic variants overall achieve lower TPRs.

In the *specific* scenario, only KS(conf) reliably detects all of the 10 out-of-specs cases. The other tests show a more diverse picture. Out of the 10 object classes used, only 5 are reliably detected by all tests. Of the remaining ones, the *mean* test misses four completely. The symmetric mean test does better, but still often fails to identify the out-of-specs situation for 2 of the classes. The log-mean test and symmetric log-mean do worse than their non-logarithmic counterparts across the board and have problems with all five classes.

Finally, in the *static* scenario, KS(conf) again identifies all out-of-specs situations, while the *mean*, *log-mean* and *symmetric log-mean* tests have severe problems. Only the *symmetric mean* test is able to recognize the out-of-specs operation in a substantial fraction of the cases (96%–97%), but it also never achieves a perfect detection rate, as KS(conf) does.

A special case is the *static* scenario using ILSVRC data. On first sight, it might not be obvious how any of the tests is able to detect this out-of-specs situation at all. After all, all individual images come from the same distribution at the training data. A test based on single samples would not be able to do better than chance level, and given that in the *static* scenario all images in a batch are identical, the statistics computed from a batch, e.g. the mean, are identical, regardless of the batch size. The answer lies in the way how the thresholds are chosen: these depend on the batch size, as for independent samples the computed statistics become more concentrated the larger the batch size. This results in a smaller interval of acceptable values, more rejected batches, and therefore a higher TPR.

*Dependence between TPR and FPR*   For single-sample tests, the trade-off between TPR and FPR is of crucial importance. It is less critical for batch-based tests, though, because for a well-defined test, increasing the batch size will increase the TPR without negatively affecting the FPR.

Figure 7 (top row) illustrates this effect on the example of the SqueezeNet classifier on AwA2 data. For very small batches ($\textsf {bs} =10$), most tests perform similarly and none of them is able to achieve high TPR and low FPR at the same time. When increasing the batch size, the TPR increases for all tests across all FPR values, with the mean test usually achieving the best results, followed closely by KS(conf). For sufficiently large batch ($\textsf {bs} =1000$), some tests are able to achieve high TPR even at very low FPR, in particular KS(conf) and, slightly below, the mean test and the symmetric mean test.

An interesting phenomenon is illustrated in the bottom row of Fig. 7: in the *static* out-of-specs situation, the selected FPR has a much weaker influence on the TPR than in the *diverse* scenario. Except for KS(conf) and the symmetric mean test, all tests have regimes where their average TPR is even below the targeted FPR. Note, however, that these occur for high FPR values, so they are not of core interest for practical applications.

*Dependence between TPR and batch size*   One insight from of our experiments is that, at a fixed FPR, the batch size needed to achieve a certain TPR depends strongly on the characteristics of the out-of-specs situation. For the sake of concreteness, we use a fixed $\text {FPR}=0.01$ in the discussion of this effect.

For the *diverse* scenarios all test reliably detect the out-of-specs situation if the batch sizes is at least 50–100 (DAVIS) or 1000–5000 (AwA2). Figure 8a and b illustrates the easiest (DAVIS, VGG19) and the most difficult (SqueezeNet, AwA2) cases. In several of the easier cases, KS(conf) require slightly larger batch sizes than some of the other tests, presumably because the other tests make implicit assumptions that are indeed fulfilled in these situations.

In the *specific* scenario, KS(conf) reliably detects all of the 10 out-of-specs cases with batches of size 50–1000. Figure 8c and d illustrates the diversity of these problems, again by displaying an easy (ResNet50, bat) and a hard case (SqueezeNet, seal).

The difference between KS(conf) and the other tests becomes most apparent in the case where the out-of-specs situation is not just due to different image data, but also due to a lack of diversity. KS(conf) reliably detects this *static* situation already at batch sizes as low as 30. All other tests, however, have problems and reach high TPR only for large batch sizes, or not at all. Figure 8e and f illustrates two examples (MobileNet25, static ILSVRC-test; NASNetAlarge, static DAVIS).

**Fig. 9:**
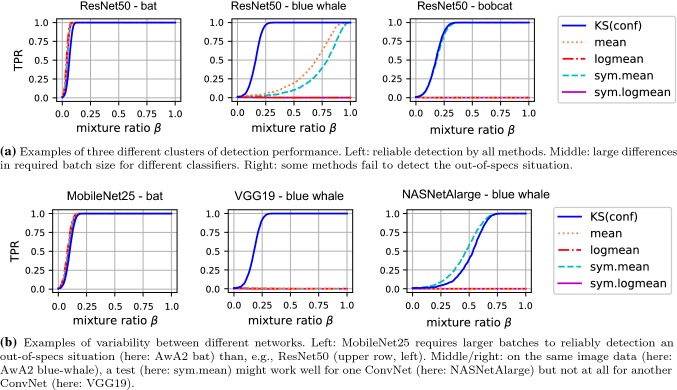
Results of detecting out-of-specs behavior with different tests for different ConvNets and data sources. *x*-axis: fraction of out-of-specs (AwA2, DAVIS) versus within-specs (ILSVRC-test) data in batch. *y*-axis: detection rate (TPR)

### Results: Fine-Grained Analysis

A main result of the previous section is that all test, except for KS(conf) , fail in some of the tested situations. To shed more light on this effect, we performed additional fine-grained experiments, where we create batches as mixtures where a fraction of $\beta$ of the images is taken from from AwA2 classes and a fraction $1-\beta$ from ILSVRC2012-test. By varying $\beta \in [0,1]$ we are able to control not only the type of out-of-specs situation, but also the strength. While, technically, all mixtures with $\beta >0$, are out-of-specs, it is clear that mixtures with smaller $\beta$ will be harder for tests to detect, if only because each batch contains fewer out-of-specs examples.

For each situation, we run all detection methods with batch size 1000 and $\text {FPR}=0.01$. The results fall into three characteristic clusters: 1) some sources, for example AwA2-bat, are identified reliably by all tests for all ConvNets, as long as the mixture proportions exceed a critical value. 2) other sources, for example AwA2-bobcat, are identified reliably by some tests, but not at all by others. 3) for some sources, here AwA2-blue-whale, tests show different sensitivities, i.e. some tests work only for high mixture proportions. Figure 9a illustrates the above examples for the ResNet50 classifier.

Interestingly, the results differ substantially not only between data sources but also between networks. For example, ResNet50 allows for perfect detection at lower mixture proportions than MobileNet25. For NASNetAlarge on blue whale data, the symmetric mean test works as least as well as KS(conf), while the same test on the same images fails completely for VGG19. An illustration of these examples is provided in Fig. 9b.

A possible explanation of the observed effects lies in the fact that score distributions differ not only strongly between ConvNets, as we had observed in Fig. 5, but also between different data sources for the same ConvNets. For example, AwA2-bat exhibits a pattern as one would ideally expect from an unknown class: confidences are overall much lower, so the difference in distribution is easy to detect for all tests. The distribution for AwA2-blue-whale data differs much less from the within-specs situation, making it harder to detect. Finally, AwA2-bobcat shows quite unexpected behavior: even though no images of this class were used at training time the networks make overall more confident predictions than for within-specs data. This is also the reason why the single-sided mean-based tests fail for this out-of-specs situation. For space reasons, we do not include illustrations of all discussed score distributions. They can be found on the accompanying website.

### Discussion of Results

Overall, our experiments show that KS(conf) works reliably in all experimental conditions we tested. This is in agreement with the expectations from theory, because the underlying Kolmogorov–Smirnov test is known to have asymptotic power 1, meaning that if given enough data, it will detect any possible difference between distributions. In contrast to this, the baseline tests show highly volatile behavior, making them unsuitable as a reliable out-of-specs detector.

On first sight, the results of *diverse* vs. *specific* vs. *static* situations might appear counter-intuitive. Intuitively, one could expect that a more specialized data distribution, e.g. all images showing the same object class, should be *easier* to detect than a more generic distribution, and for KS(conf) this is indeed the case.

For the baseline tests, however, the opposite seems to be true. The explanation lies in a bias-variance trade-off: all baseline tests essentially perform outlier detection, i.e. they trigger if a batch contains a certain number of images that would have been unlikely to occur in the with-specs situation. For a generic out-of-specs distributions, any sufficiently large batch is likely to contain sufficiently many such unexpected images, and the tests will indeed trigger quite reliably. For a highly peaked out-of-specs distribution, however, the score distribution has a higher bias and lower variance: depending on the data, is will consists either mostly of atypical images, which indeed is easily detected, or mostly of typical images, which causes the test to miss the batch. It is in this aspect where KS(conf) works fundamentally different than the other tests. As it compares the full distribution of scores, it identifies not only the situation when the scores are too low or too high, but also when the score distribution in a batch is not diverse enough.

**Fig. 10:**
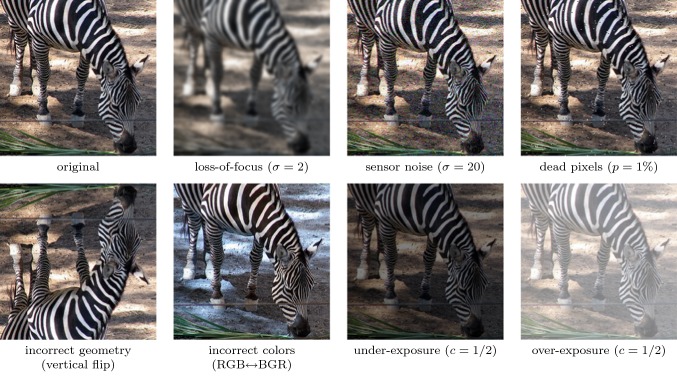
Illustration of the effect of camera system changes on the input images (Color figure online)

## Experiments: Detecting Changes to the Image Acquisition Setup

We now turn our attention to a specific form of out-of-specs operation for image classifiers: changes in the characteristics of the image acquisition setup, such as increased pixel noise due to an aging image sensor, or image blur due to a misaligned lens.

In order to study these in a quantitative way, we create new test images by applying characteristic manipulations to the images of the ILSVRC test set. Specifically, we perform the following operations. Details of their implementation can be found in the corresponding sections.

- *loss-of-focus*: we blur the image by filtering with a Gaussian kernel,
- *sensor noise*: we add Gaussian random noise to each pixel,
- *dead pixels*: we set a random subset of pixels to pure black or white,
- *incorrect geometry:* we flip the image horizontally or vertically, or we rotate it by 90, 180 or 270 degrees,
- *incorrect RGB/BGR color processing*: we swap the *B* and *R* color channel,
- *under- and over-exposure*: we scale all image intensities towards 0 or 255.

Figure 10 illustrates the operations at low to medium strength.

The main goal of our experiments in this section is not to compare the power of different out-of-specs tests, as we believe the previous sections did so in sufficient detail. Instead, we are interested in the effect itself: how exactly do different ConvNets react if their inputs change due to external effects, such as incorrect camera installation, incorrect image exposure, or broken sensor pixels? We find this a question of independent interest, with potential influence on the design of image classification system for practical tasks.

For each situation we first illustrate the changes in score distributions by three complimentary quantities: the area under the ROC curve, reflecting in how far the confidence scores of distorted images are lower than for undistorted images; the total variation distance, providing a classifier-independent measure of similarity between the distributions, and the smallest batch size at which KS(conf) with $\text {FPR}=0.01$ consistently identifies the change in all cases across 10,000 batches, serving as a proxy how hard the detection of the change is for an actual out-of-specs test.

We then highlight individual cases in more details, concentrating on the extreme or unexpected cases. As a more in-depth analysis could find further noteworthy effects, the raw data and the code of the analysis are available for public use.

**Table 5:** Distribution characteristics for a *loss of focus* in image acquisition

Loss of focus	MobileNet25			SqueezeNet			ResNet50			VGG19			NASNetAlarge		
	AUC	TV	bs	AUC	TV	bs	AUC	TV	bs	AUC	TV	bs	AUC	TV	bs
$\sigma =1$	0.56	0.09	3000	0.69	0.27	300	0.66	0.22	300	0.70	0.26	300	0.55	0.08	3000
$\sigma =2$	0.74	0.36	100	0.86	0.55	30	0.80	0.44	50	0.86	0.56	30	0.66	0.24	300
$\sigma =3$	0.85	0.53	30	0.92	0.67	30	0.87	0.57	30	0.91	0.66	30	0.75	0.39	100
$\sigma =4$	0.88	0.60	30	0.95	0.76	30	0.91	0.64	30	0.93	0.71	30	0.80	0.48	50
$\sigma =5$	0.91	0.67	30	0.97	0.80	10	0.92	0.68	30	0.95	0.76	10	0.84	0.55	30
$\sigma =6$	0.94	0.73	10	0.97	0.82	10	0.93	0.69	30	0.96	0.80	30	0.87	0.60	30
$\sigma =8$	0.96	0.80	10	0.98	0.85	10	0.91	0.65	30	0.97	0.83	10	0.92	0.68	30
$\sigma =10$	0.97	0.83	10	0.99	0.88	10	0.88	0.60	30	0.97	0.84	10	0.94	0.73	30

For each network (in columns) and blur strength, $\sigma$, (in rows), AUC denotes the area under the ROC curve between the confidences scores of the distorted and the undistorted images. TV denotes the estimated total variation distance between their distributions. bs denotes the batch size required for KS(conf) with $\text {FPR}=0.01$ to correctly report all tested batches as out-of-specs

**Fig. 11:**
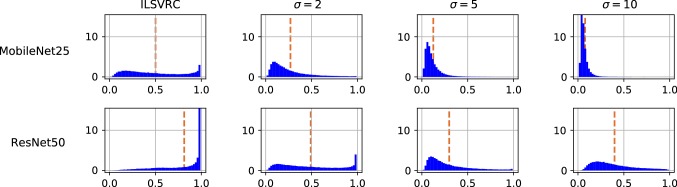
Illustration of the different reaction of MobileNet and NASNetAlarge to image blur in terms of the distribution of their confidence scores (value on *x*-axis). See Sect. 8.1 for a discussion

### Loss of Focus

To analyze how a defocussing of the camera setup influences the ConvNet outputs, we create 100,000 perturbed test images by applying a Gaussian filter with variance $\sigma$, for each $\sigma \in \{1,2,\dots ,10\}$. The filtering is performed in horizontal and vertical directions, but not across color channels.

3 $$\begin{aligned} {\tilde{X}}_t = X_t *g_{\sigma } \end{aligned}$$

for $t=1,\dots ,100000$. Note that here and in the following sections, all operations act on the original images, that is, before potential rescaling or normalization that are part of the ConvNets’ preprocessing.

Table 5 summarizes the characteristics of the resulting confidence score distributions. On the one hand, it shows the expected trend, that stronger distortions lead to stronger difference in score distribution and are easier to detect. On the other hand, what is noteworthy is a rather big difference between different networks. For some networks, such as SqueezeNet and VGG19, already a rather mild blur level, such as $\sigma =2$, leads to a substantial change of score distribution, which can be detected quite reliably even by a single-sample threshold test. In contrast, for NASNetAlarge the impact on the score distribution is much smaller, and the same level of detection quality would only be achieved by a much stronger blur of $\sigma =6$. Similarly, for batch-based testing, the necessary batch size for reliably detection differs by a factor of 10 between the both extremes. The ResNet50 shows a curious pattern: very strong blurs, $\sigma \ge 8$, become harder to detect than weaker ones, at least for single-sample tests. Figure 11 illustrates this behavior by showing the actual distribution of confidence scores. For MobileNet25, with stronger distortion, the scores become more and more concentrated around very low values. For ResNet50, the same effect happens until $\sigma =5$, though in weaker form, but for $\sigma =10$, the scores are more spread out again across all confidence values. This means in particular, that the ResNet50 will often produce a high confidence values even for very highly blurred images, while for the MobileNet25 this is almost never the case.

**Table 6:** Distribution characteristics for *sensor noise* in image acquisition

Noise strength	MobileNet25			SqueezeNet			ResNet50			VGG19			NASNetAlarge		
	AUC	TV	bs	AUC	TV	bs	AUC	TV	bs	AUC	TV	bs	AUC	TV	bs
$\sigma =5$	0.52	0.03	–	0.52	0.03	–	0.52	0.03	–	0.52	0.03	–	0.50	0.02	–
$\sigma =10$	0.56	0.08	3000	0.58	0.11	1000	0.54	0.06	5000	0.53	0.05	–	0.51	0.02	–
$\sigma =15$	0.60	0.15	500	0.64	0.20	300	0.56	0.09	3000	0.56	0.08	3000	0.51	0.03	–
$\sigma =20$	0.65	0.22	300	0.69	0.28	300	0.59	0.13	1000	0.59	0.13	1000	0.52	0.04	10,000
$\sigma =30$	0.73	0.34	100	0.73	0.35	100	0.66	0.23	300	0.67	0.24	300	0.54	0.06	3000
$\sigma =50$	0.80	0.46	50	0.76	0.40	100	0.77	0.41	100	0.78	0.42	50	0.58	0.14	1000
$\sigma =100$	0.85	0.56	30	0.81	0.57	30	0.81	0.49	50	0.82	0.48	50	0.73	0.38	100

For each network (in columns) and noise strength, $\sigma$, (in rows), AUC, TV and bs are reported as for Table 5. Missing entries in the $\textsf {bs}$ column indicate that the maximal tested batch size of 10,000 was not sufficient to reliably detect the manipulation

**Fig. 12:**
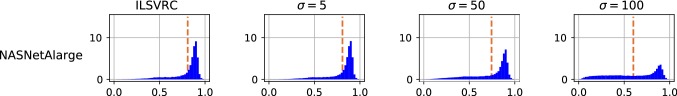
Illustration of the distribution of confidence scores (value on *x*-axis) for NASNetAlarge under different amounts of image noise. A discussion is provided in Sect. 8.2

### Sensor Noise

To analyze the effect of sensor noise, we create 100,000 perturbed test images by adding independent Gaussian noise with variance $\sigma$ in all color channels, for $\sigma \in \{5,10,15,20,30,50,100\}$:

4 $$\begin{aligned} {\tilde{X}}_t[h,w,c] = {{\,\mathrm{\texttt {clip}}\,}}_{0}^{255}\big (X_t[h,w,c] + \sigma {{\,\mathrm{\texttt {rnd()}}\,}}\big ) \end{aligned}$$

for $t=1,\dots ,100000$, where ${{\,\mathrm{\texttt {rnd()}}\,}}$ generates samples from a standard Gaussian distribution and ${{\,\mathrm{\texttt {clip}}\,}}_\text {0}^\text {255}(\cdot )$ denotes the operation of clipping a value to the interval [0, 255]. *h* and *w* range over the horizontal and vertical coordinates, respectively, and *c* over the three color channels.

The characteristics of the resulting confidence score distributions are summarized in Table 6. Similar to the out-of-focus case, for each tested ConvNet, there is an obvious trend that stronger distortions are generally easier to detect. In fact, the weakest tested noise level, $\sigma =5$, was undetectable from the score distributions for all networks. Also similar to the out-of-focus case, there are substantial differences in how strongly the different ConvNets react to the image distortions. The SqueezeNet is most susceptible, followed by the MobileNet25 and the ResNet50. VGG19 is less affected, with even $\sigma =10$ leading to almost no change in score distribution and being undetected by the batch-based test. NASNetAlarge is least affected by noise, with $\sigma =20$ being the smallest noise level that is detectable at all, and the score distribution hardly changes even up to $\sigma =50$.

Figure 12 shows the NASNetAlarge’s actual score distribution. One can see that it remains rather stable up to $\sigma =50$, and even at $\sigma =100$ a substantial amount of probability mass is still present at high confidence values.

**Table 7:** Distribution characteristics for *pixel defects* in image acquisition

Pixel defects (%)	MobileNet25			SqueezeNet			ResNet50			VGG19			NASNetAlarge		
	AUC	TV	bs	AUC	TV	bs	AUC	TV	bs	AUC	TV	bs	AUC	TV	bs
$p=1$	0.58	0.12	1000	0.66	0.24	300	0.67	0.25	300	0.60	0.14	1000	0.54	0.06	5000
$p=5$	0.67	0.26	300	0.67	0.24	300	0.73	0.34	100	0.71	0.31	300	0.59	0.14	1000
$p=10$	0.75	0.37	100	0.75	0.38	100	0.75	0.38	100	0.76	0.39	100	0.62	0.20	300
$p=20$	0.83	0.50	30	0.74	0.41	50	0.78	0.43	50	0.72	0.32	100	0.67	0.29	300
$p=30$	0.85	0.53	30	0.64	0.38	100	0.74	0.40	50	0.68	0.24	300	0.72	0.37	100
$p=40$	0.85	0.55	30	0.60	0.43	100	0.67	0.39	50	0.68	0.24	300	0.78	0.47	50
$p=50$	0.85	0.57	30	0.61	0.47	50	0.62	0.41	50	0.73	0.32	100	0.84	0.57	30
$p=60$	0.85	0.60	30	0.62	0.51	50	0.60	0.43	50	0.81	0.47	30	0.88	0.63	30
$p=80$	0.87	0.69	30	0.64	0.62	30	0.64	0.40	50	0.91	0.73	10	0.55	0.16	1000
$p=100$	0.84	0.69	10	0.66	0.71	30	0.84	0.66	10	0.92	0.76	10	0.58	0.54	50

For each network (in columns) and defect probability, *p*, (in rows), AUC, TV and bs are reported as for Table 5

**Fig. 13:**
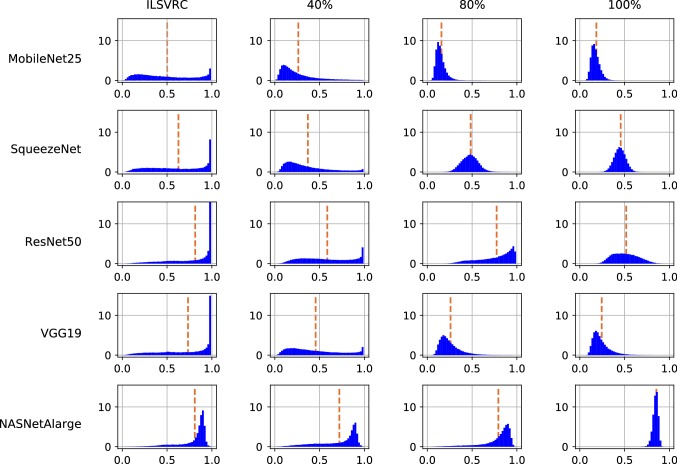
Illustration of the distribution of confidence scores (value on *x*-axis) for the give ConvNets under different amounts of pixel defects. A discussion is provided in Sect. 8.3

**Table 8:** Distribution characteristics for *under-exposure* (upper table) or *over-exposure* (lower table) in image acquisition

Exposure factor	MobileNet25			SqueezeNet			ResNet50			VGG19			NASNetAlarge		
	AUC	TV	bs	AUC	TV	bs	AUC	TV	bs	AUC	TV	bs	AUC	TV	bs
*(a) Under-exposure*															
$c=1/2$	0.53	0.04	10,000	0.61	0.15	500	0.55	0.07	3000	0.58	0.10	1000	0.50	0.02	–
$c=1/3$	0.56	0.09	3000	0.67	0.23	300	0.60	0.14	500	0.63	0.16	300	0.51	0.02	–
$c=1/4$	0.61	0.15	500	0.72	0.32	100	0.65	0.20	300	0.67	0.23	300	0.51	0.03	–
$c=1/5$	0.65	0.21	300	0.77	0.39	100	0.69	0.25	300	0.72	0.30	100	0.52	0.04	10,000
$c=1/10$	0.81	0.46	50	0.89	0.60	30	0.83	0.47	50	0.87	0.57	30	0.57	0.10	3000
$c=1/20$	0.91	0.66	30	0.93	0.70	30	0.94	0.73	30	0.97	0.81	10	0.67	0.25	300
$c=1/50$	0.89	0.71	30	0.91	0.76	10	0.99	0.91	10	0.99	0.94	10	0.90	0.62	30
$c=1/100$	0.91	0.84	10	0.89	0.84	10	1.00	0.97	5	1.00	0.97	5	0.99	0.91	10
*(b) Over-exposure*															
$c=1/2$	0.54	0.05	5000	0.71	0.30	100	0.59	0.13	1000	0.65	0.20	300	0.50	0.02	–
$c=1/3$	0.60	0.15	500	0.76	0.38	100	0.68	0.25	300	0.74	0.35	100	0.51	0.03	–
$c=1/4$	0.65	0.23	300	0.77	0.42	50	0.74	0.35	100	0.80	0.45	50	0.52	0.04	10,000
$c=1/5$	0.68	0.28	100	0.79	0.44	50	0.79	0.42	50	0.84	0.52	30	0.54	0.05	3000
$c=1/10$	0.77	0.41	50	0.87	0.57	30	0.90	0.61	30	0.93	0.70	30	0.62	0.18	300
$c=1/20$	0.91	0.66	30	0.96	0.79	30	0.95	0.76	30	0.98	0.85	10	0.75	0.39	100
$c=1/50$	1.00	0.97	5	1.00	0.98	5	0.98	0.87	10	1.00	0.96	5	0.93	0.70	30
$c=1/100$	1.00	0.98	1	1.00	0.98	1	1.00	0.97	5	1.00	0.98	5	0.99	0.93	10

For each network (in columns) and exposure factor, *c*, (in rows), AUC, TV and bs are reported as for Table 5

### Pixel Defects

We analyze the effect of *cold* and *hot* dead pixel defects by creating 100,000 perturbed test images with salt-and-pepper noise. We distort a random subset of *p* percent of the pixels, setting half of them to pure black and half of them to pure white, for $p\in \{1\%,5\%,10\%,20\%$, $40\%,60\%,80\%,100\%\}$. Formally, the operation is

5 $$\begin{aligned} {\tilde{X}}_t[h,w,c] = {\left\{ \begin{array}{ll} 0 &{} \text {if } (h,w)\in J_{\text {dead}}, \\ 255 &{} \text {if } (h,w)\in J_{\text {hot}}, \\ X_t[h,w,c] &{} \text {otherwise.} \end{array}\right. } \end{aligned}$$

for $t=1,\dots ,100000$, where $J_{\text {dead}}$ and $J_{\text {hot}}$ are disjoint random subsets of size $\lfloor \frac{1}{2}pN\rfloor$ each, where *N* is the number of pixels in the image.

Table 7 reports on the characteristics of the resulting confidence score distributions. For the MobileNet25 and, to a lesser degree, for the SqueezeNet, the expected pattern emerges that a large amount of pixel defects strongly impacts the score distribution and the corresponding out-of-specs situation can therefore be detected rather easily. For the other ConvNets, however, we observe a different pattern. For each of them, a small number of pixel defects, e.g. up to $p=10\%$, leads to a divergence of the score distribution compared to the undistorted case. This is visible in an increasing TV and AUC values, and a decreasing required batch size for KS(conf) . Higher levels, however, influence the ConvNets in different way: for ResNet50, the TV value remains almost constant between $p=10\%$ and $p=80\%$, while the AUC value declines again. This indicates that probability mass is redistributed from lower to higher confidence values, not in the other direction as one might have suspected. For example, at $p=60\%$, the AUC value is only 0.60, meaning that a threshold-based classifier would be hardly better than random at detecting this situation of heavily distorted images. For SqueezeNet, we observe the opposite effect: the AUC value remains stable over a wide range of *p*-values, while the TV value increases. For VGG19, there is range between $p=30\%$ and $p=40\%$ distorted pixels, where the TV and AUC values are lower than even at $p=5\%$. Even high levels of distortion then lead to a big change in score distribution, though, and can easily be detected. NASNetAlarge shows a similar pattern, but shifted to even higher distortion levels. Until $p=60\%$, the distribution gets more and more different from the undistorted case. At $p=80\%$, however, both the TV and the AUC values drop substantially, making this specific amount of distortion extremely hard to detect.

The observations from the numeric summaries are confirmed by the actual score distributions, a subset of which we depict in Fig. 13. It shows drastically that for all networks the score distributions fluctuate: while for undistorted images high confidences scores dominate, for higher amounts of distortion ($p=40\%$), the scores are far lower for most networks. When the number of pixel defects is very high ($p=80\%$), the scores generally increase again. For $p=100\%$, i.e. the images consist of a random arrangement of black and white pixels, for all ConvNets the score distribution is quite peaked, but not necessarily at low values. For example, for SqueezeNet and ResNet50, confidence values around 0.5 are most common, while for MobileNet25 and VGG19, the values are lower. A special case is NASNetAlarge, which changes its score distribution much less than the other networks. It is most spread out around $p=60\%$ (not depicted), but then returns to high values again for stronger distortions. For $p=80\%$ the distribution is comparable to the one for unperturbed images, and for $p=100\%$ the predicted confidence values are on average even higher than that.

**Fig. 14:**
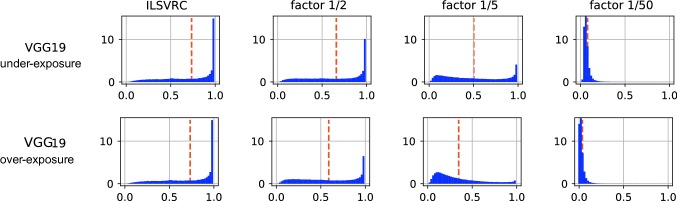
Illustration of the distribution of confidence scores (value on *x*-axis) for the VGG19 ConvNet under different amounts of under- or over-exposure. A discussion is provided in Sect. 8.4

### Under- and Over-Exposure

To study the effect of exposure changes, for each factor $c\in \{1/2, 1/3, 1/4, 1/5, 1/10, 1/20, 1/50$, $1/100\}$ we create 100,000 perturbed test images by scaling the intensity values towards 0 (under-exposure),

6 $$\begin{aligned} {\tilde{X}}_t[h,w,c]&= c \cdot X_t[h,w,c] \, \end{aligned}$$

or towards 255 (over-exposure),

7 $$\begin{aligned} {\tilde{X}}_t[h,w,c]&= 255-c\cdot (255-X_t[h,w,c]) \end{aligned}$$

for $$t=1,\dots ,100000.$$

The characteristics of the resulting confidence score distributions are reported in Table 8. For each ConvNet, it shows a conventional picture: the larger the amount of under- or over-exposure the more the resulting score distribution differs from the original one. As in previous cases, the variability between ConvNets is large, though. For SqueezeNet and VGG19, a factor of $c=1/5$ has a substantial influence and can be detected rather well. For NASNetAlarge, the score distribution for this factor is still almost indistinguishable from the situation of undistorted images. More extreme factors are then also easily detectable, though. Overall, over-exposure seems to have a bit more influence on the score distribution than under-exposure.

Figure 14 gives further insight into specific property of the score distributions, illustrated on the example of VGG19. One can see that with stronger over- or under-exposure up to $c=1/5$, the confidence scores indeed overall decrease. However, it is not a uniform change of probability mass from high to low. Instead, the original peak at high confidence scores is gradually decreased while a new peak of low confidence scores emerges. At $c=1/50$, only the peak at low values remains.

The bimodal shape of the distribution implies that even though the average confidence is low, predictions with high confidence are still quite likely to occur, at least when the network operations of images with an intermediate amount of under- or over-exposure.

**Table 9:** Distribution characteristics for problems with *geometry* or *color processing* in image acquisition

Transformation	MobileNet25			SqueezeNet			ResNet50			VGG19			NASNetAlarge		
	AUC	TV	bs	AUC	TV	bs	AUC	TV	bs	AUC	TV	bs	AUC	TV	bs
Horizontal flip	0.50	0.02	–	0.51	0.02	–	0.51	0.02	–	0.52	0.03	–	0.50	0.02	–
Vertical flip	0.68	0.27	300	0.70	0.30	100	0.70	0.30	100	0.73	0.34	100	0.62	0.21	300
$90^\circ$	0.69	0.29	300	0.70	0.29	300	0.71	0.30	100	0.74	0.35	100	0.62	0.20	300
$180^\circ$	0.68	0.27	300	0.71	0.30	100	0.70	0.30	300	0.73	0.34	100	0.62	0.20	300
$270^\circ$	0.69	0.29	100	0.70	0.30	100	0.71	0.30	100	0.74	0.35	100	0.62	0.20	300
RGB$\leftrightarrow$BGR	0.58	0.12	1000	0.60	0.14	500	0.62	0.18	300	0.62	0.17	300	0.54	0.07	3000

### Geometry and Color Preprocessing

To simulate incorrect camera installations or geometry preprocessing, we benchmark KS(conf) with horizontally and vertically flipped images, as well as images that were rotated by 90, 180 or 270 degrees. To simulate incorrect color preprocessing, we use images in which the R and B channel have been swapped. For each transformation, we create 100,000 perturbed test images.

Table 9 summarizes the characteristics of the score distribution. One can see that all of the network outputs are unaffected by horizontal flips of the training set. Presumably, the networks either learned a horizontal symmetry of the visual world, or this behavior was enforced by a data augmentation step during training. Vertical flips and rotations do have an impact on the score distribution that can be detected reliably using batches of 100 to 300 samples, while single-sample threshold classifiers would not be able to achieve high TPR or low FPR at the same time. Surprisingly, a swap of color channels does not strongly influence the score distribution. The corresponding out-of-specs situation can be detected well by a batch-based test, but with values of 300 to 3000 the necessary batch size is rather high. Tests that act on a single-sample cannot be expected to work much better than random chance for this situation.

### Discussion of Results

In summary over all experiments of this section, the main consistent aspect is that all tested ConvNets behave quite differently. A priori it is hard to predict how the outputs of a ConvNet will be affected by specific image distortions.

The two networks that target efficiency and a small memory footprint, MobileNet25 and SqueezeNet, were generally most affected by image distortions. On the one hand, this means that their predictions might become unreliable over time if they operate continuously using images from a camera system whose image quality might deteriorate over time. On the other hand, these network at least allow the reliable identification of such out-of-specs conditions in most situations.

The NASNetAlarge was consistently the least affected by the simulated changes to the camera setup. This, however, does not automatically mean that its predicted class labels are more accurate, only that their confidence scores are less useful for predicting out-of-specs situations. This distinction is particularly apparent in the case of pixel defects, where the network consistently outputs very high confidence scores even to images that consist to a large part, or even completely, of a random arrangement of black and white pixels. Of course, the predicted labels in this situation are not actually the correct object classes, but in fact all images get assigned the same ILSVRC class label window screen.

**Table 10:** Tabulated thresholds $\theta _{\alpha ,m}$ for Kolmogorov–Smirnov test

$\alpha$	$m=$ 1	3	5	10	30	50	100
0.00001	0.999995	0.982894897461	0.912933349609	0.717041015625	0.436859130859	0.342071533203	0.243988037109
0.00005	0.999975	0.970764160156	0.879882812500	0.674682617188	0.407958984375	0.319091796875	0.227416992188
0.0001	0.999950	0.963165283203	0.861999511719	0.654785156250	0.394775390625	0.308624267578	0.219879150391
0.0005	0.999950	0.937011718750	0.809631347656	0.604309082031	0.361938476562	0.282653808594	0.201263427734
0.001	0.999500	0.920654296875	0.781372070312	0.580444335938	0.346740722656	0.270690917969	0.192687988281
0.005	0.997500	0.864257812500	0.705444335938	0.518737792969	0.308166503906	0.240386962891	0.171051025391
0.01	0.995000	0.828979492188	0.668579101562	0.488891601562	0.289855957031	0.226043701172	0.160797119141
0.05	0.975000	0.707519531250	0.563232421875	0.409240722656	0.241699218750	0.188415527344	0.134033203125
0.1	0.950000	0.635986328125	0.509521484375	0.368652343750	0.217529296875	0.169616699219	0.120666503906
0.5	0.750000	0.434570312500	0.341796875000	0.341796875000	0.145874023438	0.113891601562	0.081176757812

$\alpha$	$m=\ 300$	500	1000	3000	5000	10,000	
0.00001	0.141807556152	0.110023498535	0.077911376953	0.045040130615	0.034900665283	0.024686813354	
0.00005	0.132125854492	0.102508544922	0.072586059570	0.041961669922	0.032516479492	0.023000717163	
0.0001	0.127731323242	0.099098205566	0.070171356201	0.040565490723	0.031433105469	0.022235870361	
0.0005	0.116882324219	0.090675354004	0.064208984375	0.037120819092	0.028762817383	0.020347595215	
0.001	0.111877441406	0.086791992188	0.061462402344	0.035533905029	0.027534484863	0.019477844238	
0.005	0.099304199219	0.077041625977	0.054557800293	0.031539916992	0.024444580078	0.017292022705	
0.01	0.093353271484	0.072433471680	0.051292419434	0.029659271240	0.022983551025	0.016258239746	
0.05	0.077835083008	0.060394287109	0.042778015137	0.024742126465	0.019172668457	0.013565063477	
0.1	0.070098876953	0.054397583008	0.038528442383	0.022285461426	0.017272949219	0.012222290039	
0.5	0.047241210938	0.036682128906	0.026000976562	0.015052795410	0.011672973633	0.008258819580	

## Shortcomings and Possible Improvements

While our analysis shows that KS(conf) has many desirable properties and excellent practical performance, it also has some shortcomings that we discuss in this section.

One fundamental limitation is the need to work on batches. Mathematically, KS(conf) is defined also when applied to individual images, i.e. batches of size 1. However, it is not very powerful in this setting. From Eq. (2) for $m=1$ and the thresholds in Table 10, one can see that it acts as a symmetric test with two thresholds in that case. An image is reported as outlier with FPR at most $\alpha$, if its confidence score lies in the top $\frac{\alpha }{2}$-quantile or in the bottom $\frac{\alpha }{2}$-quantile of scores observed at calibration time. Given our prior knowledge that confidence scores often do decrease rather than increase, it would be interesting to explore if an asymmetric version of KS(conf) can be derived that preserves the test’s asymptotic power but requires smaller batch sizes in general.

The other extremal situation, when KS(conf) is used with very a large batch size, is also noteworthy. Because the Kolmogorov–Smirnov test has perfect asymptotic efficiency, it will—given a large enough batch—identify any difference in distribution, no matter how small it may be. This can be considered a shortcoming, because it means that in order to avoid misdetections due to finite sampling, the validation set also has to grow. This situation can easily be avoided by keeping the batch size at a reasonable level, or a variant of KS(conf) can be derived based on the two-sample instead of one-sample variant of the Kolmogorov–Smirnov test. However, the effect shows that a test routine of arbitrarily high quality might not be practical after all. On the other hand, it might also not be desirable, as a human user will only be interested in being warned about *relevant* difference in the data distribution, not an arbitrary small one.

Another aspect that needs further study is the adaptation of KS(conf) to the situation where images within a batch are not independent, such as image sequences or videos. The classical Kolmogorov–Smirnov test is not directly applicable in this case, because the null distribution of the KS statistics for dependent data cannot easily be determined. Consequently, a more empirical version of the test, in particular with a more involved calibration phase, might be required. We plan to address this in future work.

Finally, a fundamental limitation of KS(conf) is that as a statistical test it makes the assumption of a data-generating distribution at prediction time. In some situations this assumption can be violated, e.g. when the input data can be manipulated adversarially. Specifically, KS(conf) might fail to identify that a network operates out-of-specs if the adversary has the power to manipulate every image in the batch by an image- and classifier-dependent procedure. This problem is not specific to KS(conf), though. It is clear that any test based on confidence scores can be made to fail when an adversary has the ability to tune the manipulations such that the confidence scores are preserved.

## Conclusion

In this work, we discussed the problem of detecting the situation that an image classifier runs outside of its specifications, i.e. when the distribution of data it has to classify differs from the distribution of data it was trained for. We put forward the hypothesis that it suffices to test if the classifier predicts out-of-specs, i.e. if the distribution of predicted confidence scores differs from the original one, provided a suitably strong detection method is used. We introduced such a procedure, named KS(conf), based an application of the classical statistical Kolmogorov–Smirnov test to the distribution of the confidence values of the predicted labels.

By extensive experiments we showed that single-sample tests, as they had been proposed in the literature, are fundamentally limited in their ability to identify the out-of-specs situation. Batch-based tests are more powerful, as they can leverage the additional information provided by a set of confidence values. For small batch sizes we found parametric tests, e.g. a mean test, to be competitive. However, in order to reliably identify any change in the score distribution, larger batches are required, and KS(conf) achieves the best detection performance of the tested method in this regime. Specifically, we found a batch size of 1000 sufficient to achieve 100% true positive rate for all test scenarios with a false positive rate no larger than 1%.

As a study of independent interest, we showed that different ConvNets react very differently to low-level changes of the input data, as they might be caused, for example, by changes to the image acquisition setup. On the one hand, we expect this to help practitioners in their choice of network architecture. On the other hand, we see it as a call for caution that experimental studies in this field must be thorough and broad in order to avoid the risk of overfitting to individual datasets or network architectures.

In conclusion, we hope that our work leads to more research on how to make automatic decision systems more trustworthy. To support this effort, our code and data are publicly available via the authors’ homepage.

## Footnotes

1 http://cvml.ist.ac.at/KSconf/.

2 We use the publicly available models fromhttps://github.com/taehoonlee/tensornets.
